# OCT-Derived Quantitative Measurement of Extent of Vascularization (“Zone”) in Retinopathy of Prematurity

**DOI:** 10.1016/j.xops.2025.100912

**Published:** 2025-08-13

**Authors:** David A. Sutter, Mani K. Woodward, John Jackson, Yakub Bayhaqi, Aaron S. Coyner, Shuibin Ni, Susan Ostmo, Talita T. Lima, Aaron Nagiel, Michael F. Chiang, Benjamin K. Young, Yifan Jian, John Peter Campbell

**Affiliations:** 1 Casey Eye Institute, Oregon Health & Sciences University, Portland, Oregon; 2 Chicago College of Osteopathic Medicine, Midwestern University, Chicago, Illinois; 3 HDMI – Dona Iris Public Woman’s and Maternity Hospital, Goiânia, Brazil; 4 Centro Brasilerio de Cirurgia de Olhos, Goiânia, Brazil; 5 Department of Ophthalmology, Children’s Hospital of Los Angeles, Los Angeles, California; 6 National Eye Institute, Bethesda, Maryland; 7 National Library of Medicine, Bethesda, Maryland

**Keywords:** Artificial intelligence, Retinopathy of prematurity, Ultra-widefield OCT

## Abstract

**Purpose:**

To provide a quantitative approach to the measurement of zone in retinopathy of prematurity (ROP) using ultra-widefield OCT (UWF-OCT).

**Design:**

Diagnostic accuracy study.

**Subjects:**

Infants undergoing ROP screening at Oregon Health Science University between June 2023 and October 2024, whose parents consented for research imaging.

**Methods:**

An investigational UWF-OCT captured scans from the first week of examination in which stage 1 or worse disease was noted on en face imaging in zone I, posterior zone II, or zone II, and image quality was adequate for quantitative analysis. A U-Net automatedly segmented B-scans to isolate the retina and choroid. En face images and retinal depth maps were used to manually identify the position of the optic nerve, fovea, and visualized temporal vascular border. Mean and minimum retinal arclength (RAL) was measured as the geodesic distance from the optic nerve to the vascular border. The area of vascularized retina (AVR) was estimated using the spherical cap formula, mean-RAL, and measured axial length.

**Main Outcome Measures:**

Analysis of variance and generalized estimating equations to compare OCT-derived eye-level measurements with demographics and clinical diagnosis of zone as determined by clinical assessment of en face UWF-OCT images. Area under the receiver operating characteristic curve (AUROC) for RAL compared with zone and all biomarkers at first examination as predictors of future treatment.

**Results:**

Eighty-five eyes from 52 patients met inclusion criteria. Retinal arclength and AVR were both associated with clinical diagnosis of zone and ranged from 7.2 to 17.3 mm and 40.3 to 213.1 mm^2^, respectively (*P* < 0.001 for both). The mean difference between zone I and zone II of 4.5 mm (95% confidence interval [CI]: 4.0–5.1) for mean-RAL (*P* < 0.001) and 80.9 mm^2^ (95% CI: 71.6–90.2) for AVR (*P* < 0.001). Posterior zone II was intermediate for all measurements. All measures of length and area had an AUROC >0.97 for diagnosis of zone I ROP.

**Conclusions:**

We present a framework for objective measurement of zone in ROP using UWF-OCT. This work complements prior work leveraging advances in imaging technology to bring quantitative and objective approaches to the diagnosis and classification of ROP.

**Financial Disclosure(s):**

Proprietary or commercial disclosure may be found in the Footnotes and Disclosures at the end of this article.

Retinopathy of prematurity (ROP) is a retinal vasoproliferative disease that threatens vision in premature and low birthweight infants worldwide. Retinopathy of prematurity arises when the normal centrifugal progression of retinal blood vessels is halted due to premature birth into an environment of supraphysiologic oxygen tension.^1^ It has long been recognized that zone, or the location of the vascular–avascular border on the retinal surface, has significant prognostic implications, with more posterior zone being associated with higher risk of ROP requiring treatment and adverse outcomes.

The International Classification of ROP (ICROP) describes zone in 4 ordinal categories: I, posterior zone II (PII), II, and III.^1^ The border of zone I is defined as a circle with a radius twice the distance from the optic nerve to fovea (foveal distance [FD]), centered on the optic nerve, and an eye is classified as zone I if any portion of the vascular border (even just a notch) is within this circle. Accurate identification of zone I eyes is critical as these eyes are at the highest risk.^1^ Zone II is the area anterior to zone I to a circle with radius of the distance from the disc to the nasal ora serrata, and zone III is the remaining crescent of temporal retina (and not usually visible on widefield digital retinal imaging). Posterior zone II is defined as the region extending 2 disc diameters from the zone I to II border and was added by ICROP 3 to acknowledge that even within zone II, the risk is higher with more posterior vascularizatoin.^1^ Despite clear definitions, in practice, the classification is made subjectively either with indirect ophthalmoscopy or fundus photography. Previous studies have shown significant interexpert variability in the identification of the fovea on photographs and diagnosis of zone on both photographs and ophthalmoscopy, which can lead to discrepancies in ROP diagnosis and treatment.^2^^,^^3^

With advances in ophthalmic imaging, most recently ultra-widefield (UWF)-OCT, it is possible not only to classify zones I to III using UWF imaging but, in theory, to measure quantitatively the anatomic corollary of zone, the area of vascularized retina (AVR). In this study, we calculated the AVR using the geodesic distance (shortest path between 2 points along a curved surface) from the optic disc to the vascular–avascular border on 3-dimensional UWF-OCT images.

## Methods

This study was conducted at the Oregon Health & Science University, abided by the tenets of the Declaration of Helsinki, and was approved by the Institutional Review Board at Oregon Health & Science University. Parents of all infants included in this study provided informed written consent.

### Data Set and Demographics

Between June 2023 and October 2024, all infants who were eligible for ROP screening at the Oregon Health & Science University neonatal intensive care unit were imaged with an investigational 800-kHz, 140-degree field of view, swept-source UWF-OCT.^4^ Before imaging, infants’ eyes were pharmacologically dilated, a lid speculum was placed, and ophthalmic lubricating gel was applied as a coupling agent. Retinopathy of prematurity physicians positioned the UWF-OCT and recorded scans. In addition to clinical demographics (e.g., birthweight [BW], gestational age [GA], postmenstrual age) and treatment status, UWF-OCT en face images were graded for zone (I–III), stage (1–5), and plus disease (no plus, pre-plus, or plus) by clinicians (J.P.C. and B.K.Y.) using ICROP 3 guidelines.^1^ From each eye that developed at least stage 1 ROP within zone I, PII, or II, we selected the earliest acceptable quality image in which the optic disc and maximal temporal retina could be visualized. If image quality precluded analysis, an image acquired within 1 week was used if zone did not change within this period.

### OCT-Based Measurements

**Figure 1:**
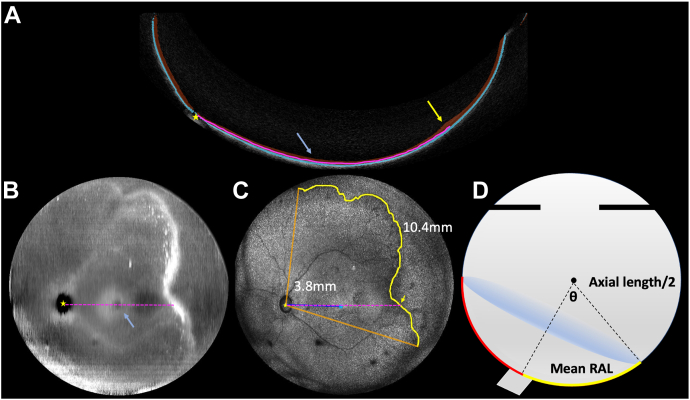
Methodology for calculation of retinal arclength (RAL) and area of vascularized retina (AVR). **A,** B-scan with retina (red) and choroid (blue) segmented. Retinal arclength is the path between the retina and choroid, along the retinal pigment epithelium (pink dotted line) from the optic nerve (yellow star) to an arbitrary point on the vascular border (yellow arrow/line). **B,** Retinal depth map (derived from retinal segmentation in A) highlighting thicker sections in white and thinner sections in black, with fovea (blue arrow) and optic nerve (yellow star) labeled. **C,** Ultra-widefield OCT (UWF-OCT) en face with all available temporal vascular border (yellow line inscribed by orange lines). The yellow arrow in A is represented as a yellow arrow in C. The foveal distance was measured at 3.8 mm, and the mean-RAL was 10.4 mm averaging 10 measured RALs per clock hour. **D,** Cross section of spherical cap diagram. All vascularized retina surface area (red) from measured mean-RAL (yellow), where the eye angle theta is equal to mean-RAL/axial length.

The axial length (AL) of each eye was calculated from the UWF-OCT axial distance to the fovea, and B-scans were segmented for the retina and choroid using a previously trained U-Net (Fig 1A).^5^ The optic nerve center, foveal center, and vascular–avascular retinal border were identified using retinal thickness maps (Fig 1B) and en face maximum intensity projections (Fig 1C). The 3D coordinate position of the underlying retinal pigment epithelium (RPE) was identified (the RPE was chosen instead of the retinal surface, as its height does not change with more severe disease). For the optic disc, the equivalent z-axis position of the RPE was calculated from the RPE position at the optic nerve edge. Next, using Bresenham’s line nearest neighbor algorithm, we connected these points and identified the centroid in 3D space. We then made retinal arclength (RAL) measurements, defined as the geodesic distance (shortest curvilinear distance between 2 points) from the optic nerve centroid to a point on the vascular–avascular border. Retinal arclengths were calculated to 10 evenly spaced points per clock hour, along all available vascular–avascular border in the temporal hemisphere. From this array of RALs we calculated both the minimum RAL (min-RAL) and mean (mean-RAL) distance, given that clinical diagnosis has always been based on the most posterior zone border, but UWF-OCT enables us to more precisely measure the retinal surface across multiple degrees. For example, in Figure 1A and 1C, a pink dotted line represents the RAL from 2 perspectives. In Figure 1A, this RAL separates the retinal and choroid segmentation along a geodesic surface, and in Figure 1C, this same RAL is viewed in 2 dimensions as a line.

Finally, the AVR was estimated using the spherical cap surface area formula, with mean-RAL and AL as the approximate diameter of the eye (Fig 1D):

$$Area\;of\;Vascular\;Retina=\pi \frac{AL^{2}}{2}\left(1–\cos\left(\frac{\bar{RAL}}{AL}\right)\right)$$

### Statistical Analysis

Statistical analysis was performed using R (4.3.1). All tests were 2-sided, and significance was determined at *P* < 0.05. Analysis of variance tests were used to determine if differences existed between zone and each clinical demographic. For these tests, one eye from each patient was randomly selected because zone presented bilaterally in all patients but one (this data was also used to compare mean-RAL to min-RAL via Pearson’s correlation coefficient). To determine whether significant differences occurred between each OCT-based measurement and zone while accounting for inter-eye correlations, generalized estimating equations with exchangeable correlation structures were used. An omnibus test was obtained by comparing each model to a null model via a Wald test.

We calculated the areas under the receiver operating characteristic curves (AUROCs) for GA, BW, and OCT-derived quantitative measures of zone for the diagnosis of zone I ROP, as well as the prediction of future type 1 ROP. For patient-level data (i.e., GA, BW), the randomly selected eye from above was used. For eye-level data, both eyes were measured via cluster-based bootstrapping (clusters [patients] were sampled with replacement 2000 times) to account for inter-eye correlations. Each predictor was estimated for each bootstrap replicate to estimate 95% confidence intervals (CIs). The mean difference between each predictor pair was used to determine significance.

## Results

### Data Set and Demographics

Of the 190 eyes from 95 infants screened for ROP during the study period, 85 eyes from 52 patients met inclusion criteria. Seventy-four eyes from 37 infants either did not develop ROP or had ROP in zone III. Two eyes from 2 patients were excluded because they were transferred in from an outside hospital with treatment-requiring ROP. In 29 eyes, although ROP diagnosis could be made from the image, the image quality was judged to be too poor for quantitative analysis. In general, infants with zone I ROP had lower GA and BW, developed ROP at an earlier postmenstrual age, and were more likely to eventually require treatment for ROP: 21 of 34 (61.8%) of zone I eyes, 3 of 9 (33.3%) in posterior zone II (PII), and none in zone II (Table 1).

**Table 1:** Demographics of Eyes at First Diagnosis of Retinopathy of Prematurity

Demographics	Zone I	Posterior Zone II	Zone II	*P*
Patients	18 (34.6)	5 (9.6)	29 (55.8)	
Eyes	34 (40.0)	9 (10.6)	42 (49.4)	
Patients requiring treatment	11 (61.1)	2 (40.0)	0 (0.0)	<0.001
Eyes requiring treatment	21 (61.8)	3 (33.3)	0 (0.0)	<0.001
Gestational age, wks	24.1 ± 1.2	24.2 ± 1.0	27.8 ± 2.1	<0.001
Birthweight, g	557.9 ± 116.8	605.0 ± 84.4	964.6 ± 414.7	<0.001
Postmenstrual age, wks	33.3 ± 1.5	32.6 ± 1.5	35.9 ± 3.1	<0.001

Values are *n* (%) or mean ± standard deviation.

### OCT-Based Measurements

Table 2 summarizes the OCT-based measurements of all the eyes in the study. The mean assessed angle of the ridge was 70.0° ± 24.3° for all eyes but higher in zone I than zone II eyes (81.6° ± 26.5° vs 62.6° ± 19.7°, *P* < 0.007). There were no measured differences in mean FD between zone classifications. For all other measures of curvilinear length (mean- and min-RAL), and area (AVR using mean-RAL), we found differences across zones I, PII, and II. We also used the mathematical definition of zone I, defined as the ratio of the most posterior vascular border (min-RAL) compared with the disc to FD and converted that into a ratio as an interpretable and continuous measure of “zone.”

**Table 2:** OCT-Based Measurements by Clinical Diagnosis of Zone

Ocular Findings	Zone I	Posterior Zone II	Zone II	*P*
AL, mm	15.4 ± 0.5	15.2 ± 0.6	16.0 ± 0.6	<0.001
FD, mm	4.5 ± 0.5	4.8 ± 0.6	4.7 ± 0.6	0.040
mean-RAL, mm	10.3 ± 1.2	11.9 ± 1.4	14.8 ± 1.1	<0.001
min-RAL, mm	8.8 ± 1.1	10.4 ± 1.2	13.6 ± 1.3	<0.001
min-RAL:FD ratio	2.0 ± 0.3	2.2 ± 0.4	3.0 ± 0.5	<0.001
AVR, mm^2^	80.7 ± 17.7	106.4 ± 23.8	161.6 ± 22.8	<0.001

AL = axial length; AVR = area of vascularized retina; FD = foveal distance; min-RAL = minimum RAL; RAL = retinal arclength.

Values are mean ± standard deviation.

**Figure 2:**
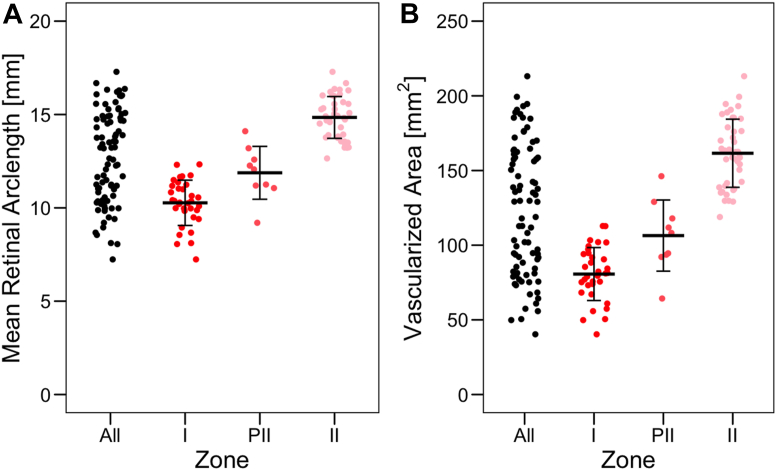
OCT-based measurements of mean retinal arclength (RAL) and area of vascularized retina (AVR) by zone. Both the (**A**) RAL and (**B**) AVR increased with each change in clinical diagnosis of zone (*P* < 0.001 for all). Plots illustrate the raw data, with horizontal lines as the mean for each zone and vertical lines as one standard deviation in either direction. PII = posterior zone II.

Figure 2 shows the distribution of mean-RAL (mm) and AVR (mm^2^) overall and across zone classifications. The mean difference between zones I and II was 4.5 (95% CI: 4.0–5.1) mm for mean-RAL (*P* < 0.001) and 80.9 (95% CI: 71.6–90.2) mm^2^ for AVR (*P* < 0.001). Additionally, measurements for mean-RAL and AVR between eyes with zone I or zone II ROP separated perfectly: for zone I and zone II eyes, the range of mean-RAL was 7.2 to 12.3 mm and 12.7 to 17.3 mm, and the range of AVR was 40.3 to 112.9 mm^2^ and 118.9 to 213.1 mm^2^, respectively.

### Diagnostic and Prognostic Value of OCT-Based Measurements

**Figure 3:**
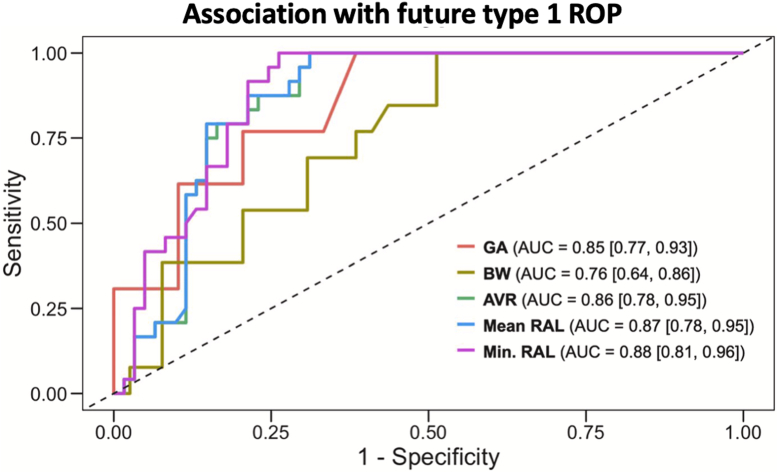
Prognostic value of birthweight (BW), gestational age (GA), and OCT-derived quantitative measurements of zone at first diagnosis of retinopathy of prematurity (ROP) for the development of treatment-requiring ROP. Area under the receiver operating characteristic curve (AUC) for prediction of future type 1 ROP for mean and minimum retinal arclength (RAL), area of vascularized retina (AVR), BW, and GA demonstrate that OCT-derived measures are at least as predictive as BW and GA (see Table S1).

The AUROC for the diagnosis of zone I ROP was 0.97 (95% CI: 0.94–1.00), 0.97 (95% CI: 0.95–1.00), and 0.97 (95% CI: 0.94–1.00) for mean-RAL, min-RAL, and AVR, respectively. Figure 3 demonstrates the prognostic association of both demographics and OCT-derived biomarkers for prediction of future type 1 ROP. We found the OCT-derived measurements were highly predictive of future type 1 ROP, at least if not better than BW and GA (Table S1, available at www.ophthalmologyscience.org). Figure S1 (available at www.ophthalmologyscience.org) demonstrates the correlation between mean-RAL and min-RAL for all eyes across zones (Pearson’s correlation coefficient = 0.96; 95% CI: 0.94–0.98).

## Discussion

This study evaluated the association of OCT-derived quantitative measures of RAL and approximated surface area (AVR) compared with the clinical diagnosis of zone. The key findings were as follows: (1) OCT-derived measures of both RAL and surface area were associated with the ordinal categories of zone (I, PII, II); (2) the underlying anatomic measurements for both arclength and area are both continuous and highly diagnostic of zone I disease; and (3) these measures at first examination with ROP were as prognostic for future type 1 ROP as demographics such as BW or GA.

The diagnosis of ROP severity has always been subjective and limited by poor interobserver agreement both on ophthalmoscopy and fundus photography.^6^^,^^7^ For zone, this is in part due to the imprecision of identifying the true foveal center and, by extension, accurately estimating the ratio to the vascular border, and previous work has demonstrated intraobserver agreement to be <70%.^2^^,^^6^^,^^8^ Without truly objective diagnosis of zone, all prior “accuracy” studies are limited by the absence of a true gold standard. Efforts to make zone classification more objective have used fundus photography and estimating distance using either a “pixel to millimeter” approximation (which depends on AL, among other things) or a disc diameter approximation (which assumes uniform size of the optic disc).^9^, ^10^, ^11^, ^12^, ^13^ There are several potential sources of error. First, experts may use other anatomic information besides the apparent location of the fovea when making the determination of zone (similar to prior work demonstrating that experts often use information other than the absolute level of dilation and tortuosity to diagnose plus disease).^14^^,^^15^ Second, it may be that approximating retinal distance based on a constant pixel/millimeter conversion may not be an accurate approximation as features are distorted nonlinearly in the periphery due to ocular curvature. In this study, we used objective data from UWF-OCT to overcome prior limitations. We precisely identified the foveal location and directly measured the RAL between the optic nerve and the most posterior retinal vascular border (min-RAL) and the mean distance to the border (mean-RAL) using the actual AL and retinal curvature. These measures were strongly associated with the clinical diagnosis of zone (AUROC 0.97 for all) and may represent an objective method of improving diagnostic consistency in the future.

All components of ROP diagnosis require clinicians to assign ordinal labels to underlying disease characteristics that appear along a continuum.^1^ Previous work analyzing expert diagnosis of plus disease led to ICROP recognition that plus disease is a spectrum and development of a novel numerical (1–9) scoring system to more precisely assess the spectrum of disease.^1^^,^^16^, ^17^, ^18^ Subsequent analysis by the ICROP committee found the same spectrum pattern for stage, and prior work using UWF-OCT has demonstrated the potential for quantitative assessment of ridge volume as an OCT biomarker for the stage spectrum in the future.^7^^,^^19^ In this article, we demonstrated that OCT-derived quantitative measures of retinal length and area are continuous, suggesting that UWF-OCT may facilitate more granular, precise, and objective assessment of retinal “zone” than current diagnostic methods allow. In addition, it may emerge that longitudinal changes in OCT-derived length/area measurements are useful for disease monitoring and prognostication as well.^20^

These data highlight that the way disease is classified helps inform our clinical decision making, for better and worse. The CRYO-ROP study first demonstrated the prognostic significance of zone I ROP, which has led to a pattern of binary thinking focused on determining whether any portion of the ROP ridge falls within a circle twice the radius of the FD.^21^ Since the early treatment for ROP study, this distinction (which may be only a pixel or 2 difference on fundus photography) can even lead to treatment differences in the case of stage 3 ROP without plus disease.^22^ The recent ICROP update added zone PII as an intermediate category to highlight the reality that the risk is much more of a continuum, but it did not change the current treatment guidelines that have separate criteria for “zone I” disease.^1^ These data further highlight a potential explanation for that clinical reality, given zone II eyes have twice the vascular area (or half the avascular area) of zone I eyes, on average. If avascular retina is a primary source of VEGF, then it follows that more avascular retina portends higher risk.^23^, ^24^, ^25^ None of the eyes in this cohort that first developed ROP in zone II required treatment. Future work can evaluate the relative (and combined) importance of OCT-derived risk factors with known and emerging risk factors, such as oxygen exposure (which is also highly correlated with GA) informs our understanding of ROP physiology.^26^^,^^27^

There are several limitations of this study. The study design compared images at first diagnosis of ROP, which was different in zone I and II eyes, meaning that zone II eyes were slightly larger (with larger area) given they were older when stage 1 developed. It also highlights the limitation that we only analyzed eyes with a clear demarcation line (stage ≥1) for reliability in segmentation of the vascular border, but future work using OCT angiography may lead to the ability to quantify RAL and AVR for all eyes with immature retina, not just the subset with stage 1 or worse ROP, and may highlight earlier stages/findings that are currently subclinical.^4^ We also had to exclude a small number of images of patients who clearly had stage 1 or worse ROP on en face OCT images but were of insufficient quality to consistently measure RAL or AVR. In most of those cases, we were able to use an image from the next week (none of which had a different clinical diagnosis of zone and there was no difference in future rates of type 1 or less ROP between those who had usable vs unusable data). In addition, we only analyzed the temporal vascular border, even though zone can be defined by either nasal or temporal border (whichever is more posterior), and previous studies have demonstrated nasal:temporal asymmetry.^28^ Future work can compare the relative prognostic importance of nasal versus temporal RAL and evaluate all 360° of retinal vascularization. Another potential limitation is that the assessed angle was higher for more posterior zone, as has been reported previously with prior methods^29^; however, this did not seem to affect the diagnostic accuracy (AUROC 0.97 for all measures) in this study. Future work could evaluate whether including nasal, superior, and inferior borders has other diagnostic or prognostic implications. Finally, the clinical diagnosis of zone in this article was based on a single examiner’s assessment at the bedside using, at least in part, the UWF-OCT images rather than a fully independent assessment using indirect ophthalmoscopy or fundus photographic documentation alone.

In this study, we present a quantitative framework for objective measurement of zone in ROP using UWF-OCT. This work complements prior work leveraging advances in imaging technology, including artificial intelligence and OCT, to bring quantitative and objective approaches to the clinical diagnosis of ROP. In the future, automated methods of ROP diagnosis may play a role as both assistive diagnostic and autonomous screening tools, which, if integrated into sustainable care delivery models, could reduce the number of babies going blind from ROP worldwide.
