# Supplementary material for: OCT-Derived Quantitative Measurement of Extent of Vascularization (“Zone”) in Retinopathy of Prematurity
Source: Ophthalmol Sci. 2025 Aug 13;6(1):100912. doi: 10.1016/j.xops.2025.100912 (PMC12548099; doi:10.1016/j.xops.2025.100912)
Supplement: Figure S1 [file mmc1.docx]

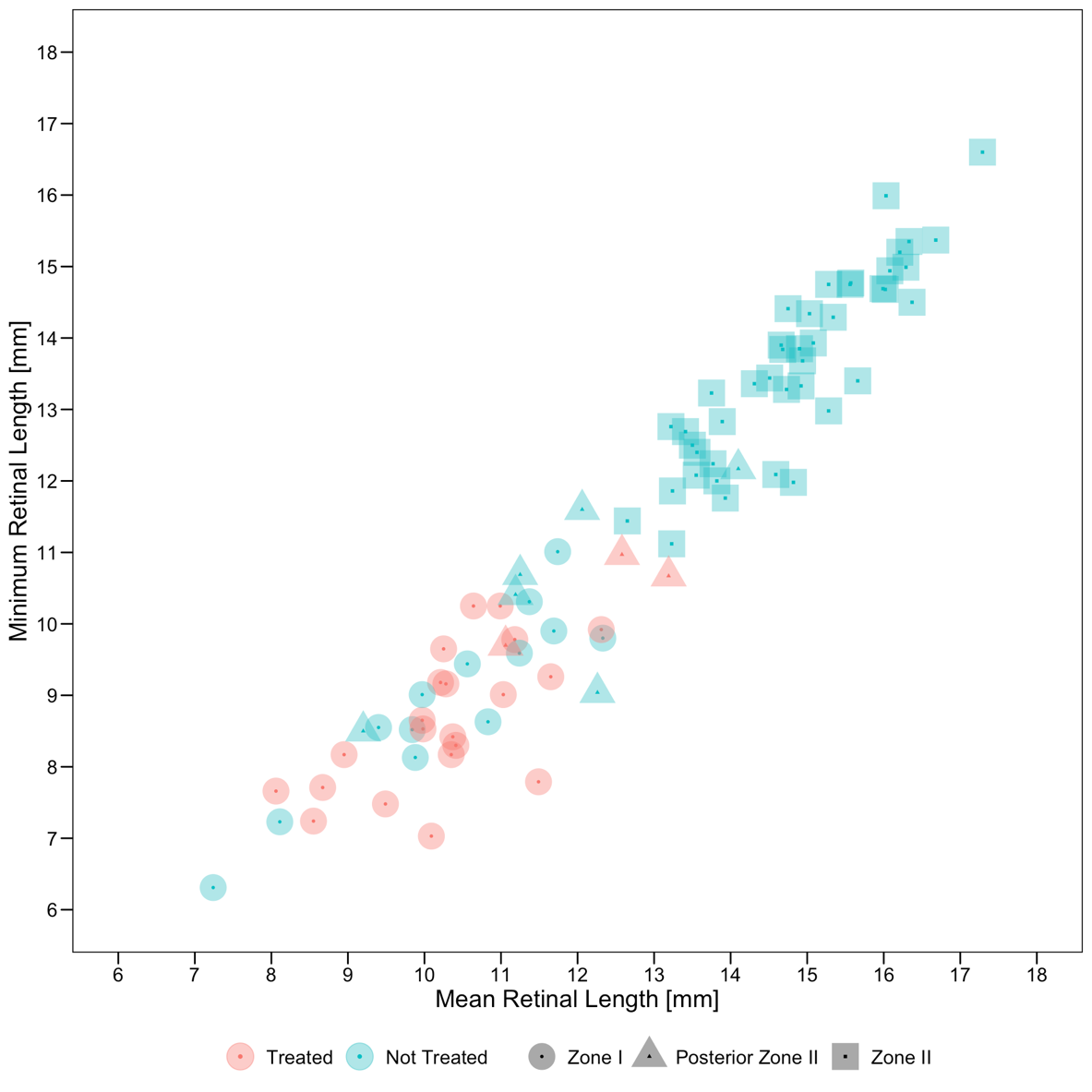


**Supplemental Figure S1: Comparison of mean and minimum retinal arclength (RAL) compared to clinical diagnosis of zone and future treatment status.** Scatterplot of min-RAL and mean-RAL demonstrating high correlation (Pearson correlation coefficient [95% CI] = 0.96 [0.94,0.98]) and clinical diagnosis of Zone I (circle), PII (triangle), II (square), categorized by treatment-requiring (red) and non-treatment-requiring (green) outcomes.
