# Supplementary material for: OCT-Derived Quantitative Measurement of Extent of Vascularization (“Zone”) in Retinopathy of Prematurity
Source: Ophthalmol Sci. 2025 Aug 13;6(1):100912. doi: 10.1016/j.xops.2025.100912 (PMC12548099; doi:10.1016/j.xops.2025.100912)
Supplement: Table S1 [file mmc2.docx]

**Supplemental Table S1: Comparison of AUROC predictive significance in demographic versus ocular findings for type-1 ROP.**

| **Comparison** | **Mean [95% CI] AUROC Difference** | **Significant** |
| --- | --- | --- |
| BW — GA | -0.10 [-0.19, -0.01] | * |
| BW — mean-RAL | -0.11 [-0.21, 0.00] |  |
| BW — min-RAL | -0.13 [-0.23, -0.02] | * |
| BW — AVR | -0.11 [-0.21, 0.00] |  |
| GA — mean-RAL | -0.01 [-0.10, 0.07] |  |
| GA — min-RAL | -0.03 [-0.11, 0.05] |  |
| GA — AVR | -0.01 [-0.10, 0.07] |  |
| Mean-RAL — min-RAL | -0.02 [-0.05, 0.02] |  |
| Mean-RAL — AVR | 0.00 [0.00, 0.01] |  |
| Min-RAL — AVR | 0.02 [-0.01, 0.05] |  |
| *****GA: gestational age at birth, BW: birthweight, RAL: retinal arclength, AVR: area of vascularized retina  **p* < 0.05 | | |
